# Porcine Circovirus type 2 (PCV2) causes apoptosis in experimentally inoculated BALB/c mice

**DOI:** 10.1186/1746-6148-1-7

**Published:** 2005-10-31

**Authors:** Matti Kiupel, Gregory W Stevenson, Elizabeth J Galbreath, Adam North, Harm HogenEsch, Suresh K Mittal

**Affiliations:** 1 Animal Disease Diagnostic Laboratory, Purdue University, West Lafayette, Indiana, 47906-1175, USA; 2 Department of Veterinary Pathobiology, Purdue University, West Lafayette, Indiana, 47906, USA; 3 Lilly Research Laboratories, Indianapolis, Indiana 46285, USA

## Abstract

**Background:**

We have previously described microscopic and electron microscopic alterations in lymphoid organs of PCV2 inoculated mice as apoptosis. In this study we wanted to investigate the molecular pathogenetic mechanism of PCV2-induced apoptosis. Eight-week old BALB/c mice were either sham inoculated (control mice) or inoculated intraperitoneally (ip) and intranasally (in) with a single (sPCV mice) or multiple (mPCV mice) doses of PCV2. Four control mice and 4 sPCV mice were sacrificed 7, 14, 28 and 42 days post inoculation (PI). All 4 mPCV mice were sacrificed 42 days PI. Following necropsy, immunohistochemistry for caspase 3 and in-situ TUNEL assay were performed on sections of spleen, lymph nodes, thymus and ileum from control, sPCV and mPCV mice. In addition, total RNA was extracted from spleens of control, sPCV and mPCV mice for simultaneous detection and semiquantitation of bcl-2 homologues and various caspase mRNAs using a multiprobe RNase protection assay system.

**Results:**

PCV2 replicated and was associated with apoptosis in spleens, lymph nodes and Peyer's patches of infected BALB/c mice. Upregulation of caspase 1, 2, 3, 6, 7, 8, 11 and 12 and upregulation for the transcripts of apoptosis inhibitors bcl-2, bcl-w and bcl-X and apoptosis promoters' bax, bak and bad was detected in spleens of sPCV and mPCV mice, but not control mice. Apoptosis was further confirmed by light and electron microscopic morphology as well as by positive TUNEL assay and detection of activated caspase 3. PCV2 nucleic acid was detected by in-situ hybridization in the nuclei and cytoplasm of such apoptotic cells.

**Conclusion:**

The data presented here support the hypothesis that PCV2 induces apoptosis mediated through the activation of caspases 8 and 3 in the spleens of infected mice.

## Background

Circoviruses, the smallest animal DNA viruses known so far, have a single copy of circular single-stranded ambisense DNA genome that varies in size between 1.7 and 2.3 kb. Animal circoviruses have been demonstrated in chickens (chicken anemia virus, ChAV, [49]), pigs (porcine circovirus, PCV, [45]), pigeons (pigeon circovirus, [47]) and psittacines (psittacine beak and feather disease virus, PBFDV, [36]). Porcine circovirus (PCV), an approximately 17 nm in diameter, non-enveloped virus with icosahedral symmetry, was originally identified as a noncytopathic contaminant of the PK-15 porcine kidney cell line [44]. The genome of PK-15 derived virus has been sequenced [28] and isolates of PCV that are genetically like PK-15 cell PCV are referred to as PCV1 [29]. Inoculation studies in pigs using PK-15 derived PCV1 did not result in clinical disease [1,46]. In 1990's, field strains of PCV have been found in lesions of pigs with postweaning multisystemic wasting syndrome (PMWS) [2,5,6,10,17,31,33,42]. Isolates of PMWS-associated PCV are genetically and antigenically different from the PK-15 cell PCV and are referred to as PCV2 [29].

PMWS is clinically characterized by progressive weight loss, dyspnea, tachypnea and less frequent diarrhea, pallor and icterus in pigs [5]. Gross lesions in pigs with PMWS consist of generalized lymphadenopathy in combination with less frequent lesions in the lungs, liver, kidneys and stomach [5,16]. The most consistent microscopic lesions in affected pigs are in lymphoid organs and include lymphoid cell depletion and granulomatous inflammation with inconsistently occurring intracytoplasmic viral inclusion bodies in macrophages [5,10,17,31,40]. PCV nucleic acid and antigen have been demonstrated within lesions in multiple organs of naturally diseased pigs with PMWS [5,6,10,18,31,40]. So far, isolates of PCV from pigs with PMWS have been identified nearly exclusively as PCV2 [2,14,15,29,31]. However, the role of PCV2 in PMWS remains unclear. PCV2 infection alone produces asymptomatic infection in germ-free pigs without evidence of overt PMWS [21]. In contrast, coinfection of PCV2 with porcine parvovirus (PPV) or concurrent injection with keyhole limpet hemocyanin in incomplete Freund's adjuvant enhanced replication of PCV, and caused PMWS [11,19,21,22].

On the basis of histopathological changes in naturally and experimentally infected pigs, it appears that PCV2 induces apoptosis in pigs in vivo. Hepatic disease has been implicated as the major cause of icterus, wasting and death in naturally occurring and experimentally reproduced cases of PMWS [21,22,39]. The predominant hepatic lesion has been described as single cell necrosis [3,11,21,22] or apoptosis [39] of hepatocytes. Only recently, ORF3 of PCV2 has been shown to play a major role in the induction of virus-induced apoptosis through activation of caspase-8 and caspase-3 pathways, but not caspase-9 [24]. However, ORF3 is not essential for viral replication and recent studies indicate that apoptosis is not a remarkable feature in PMWS lymphoid lesion development [38]. On the contrary, when assessing the proliferation/apoptosis ratio to determine cell turnover, decreased cell proliferation and not increased apoptosis was concluded to be the most important variable leading to cell depletion in PMWS lymphoid tissues [25].

We demonstrated the replication of PCV2 in 8-week old BALB/c mice following experimental inoculation [20]. In mice PCV2 caused only mild microscopic lesions in lymphoid organs characterized by proliferation and morphologic evidence of apoptosis of cells in germinal centers and mild lymphoid depletion of the paracortex. PCV2 nucleic acid was detected in the nuclei and cytoplasm of apoptotic cells in germinal centers.

The aim of this study was to confirm the described microscopic and electron microscopic alterations in lymphoid organs of mice as apoptosis and to study the molecular pathogenetic mechanism of PCV2-induced apoptosis.

## Results

### PCV2 induces apoptosis in lymphoid organs of BALB/c mice

**Figure 1:**
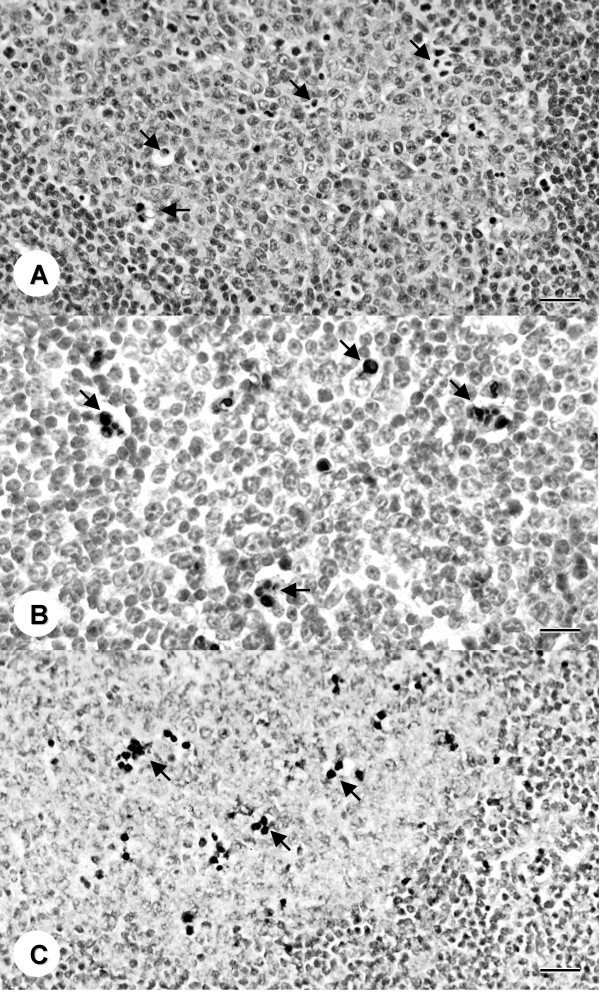
Spleen from a PCV2-inoculated sPCV mouse 28 days PI. Large numbers of apoptotic cells (arrows) in germinal centers. H & E staining, Bar = 40 μm. Same germinal center in spleen as in figure 1. Apoptotic cells (arrows) stain dark brown with TUNEL. In-situ hybridization, hematoxylin counterstain, Bar = 20 μm. Same germinal center in spleen as in figure 1. The majority of apoptotic cells (arrows) stain dark blue for PCV2 nucleic acid. In-situ hybridization, hematoxylin counterstain, Bar = 60 μm

**Figure 2:**
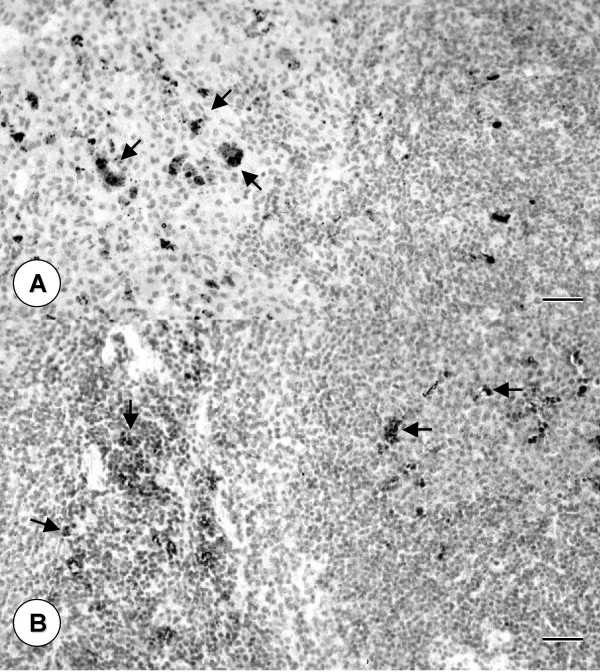
Same germinal center in spleen as in figure 1. Apoptotic cells (arrows) stain dark brown for active caspase 3. Immunohistochemistry, hematoxylin counterstain, Bar = 80 μm. Spleen from a PCV2-inoculated sPCV mouse 28 days PI. Large numbers of apoptotic cells (arrows) in sinusoids and germinal centers stain dark brown with TUNEL. In-situ hybridization, hematoxylin counterstain, Bar = 100 μm

PCV2-inoculated mice, but not control mice, had enlarged germinal centers in lymphoid tissues that were composed of cells morphologically typical of lymphoblasts and histiocytes. Numerous apoptotic cells were detected in spleen, lymph nodes and Peyer's patches of PCV2-inoculated mice but not control mice by concurrent positive TUNEL assay and immunostaining for activated caspase 3. Apoptosis was further confirmed by electron microscopy as previously described [20]. Apoptotic cells were detected in enlarged germinal centers of spleens and lymph nodes (Figures 1a–c and 2a) of sPCV mice on days 7, 14, 28 and 42 PI and mPCV mice on day 42 PI. In addition, many apoptotic cells were found in the sinusoids of the spleen (Figure 2b) of the sPCV mice at 28 days PI. There were few apoptotic cells in spleens of sPCV mice on day 7 PI. The number of apoptotic cells was significantly increased on day 14 PI and remained the same on days 28 and 42 PI in sPCV mice and on day 42 PI in mPCV mice. Apoptotic cells, identified by activated caspase-3 and TUNEL corresponded to cells with microscopic changes typical of apoptosis (Figure 1b and 2a). The cytoplasm and less frequently the nuclei of many apoptotic cells in germinal centers were positive for PCV nucleic acid by in-situ hybridization (Figure 1c). However, no PCV nucleic acid was identified in apoptotic cells in the sinusoids of the spleen of sPCV mice at 28 days PI.

### PCV2 increases mRNA of caspases in spleens of BALB/c mice

**Figure 3:**
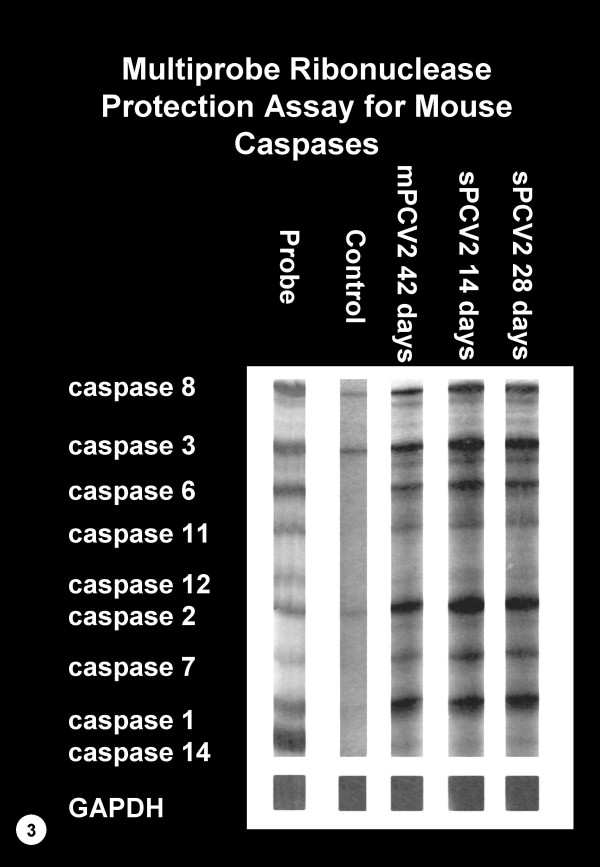
RNAse protection assay (RPA) for various caspase mRNAs from spleens of individual BALB/c mice. The probe and a representative RPA of a control mouse 28 days post sham inoculation are shown on the left. RPAs for representative BALB/c mice that were inoculated with a single (sPCV2) or multiple (mPCV2) doses of PCV2 and were euthanized at 14, 28 or 42 days PI, are shown on the right. Transcripts for caspases 1, 2, 3, 6, 7, 8, 11 and 12 are increased in sPCV and mPCV mice when compared to control mice. Upregulation of caspases 1, 2, 3, 6 and 8 are strongest and bands for caspase 11 and 12 are only faint. Quantitation of mRNA is identical for all samples.

RPAs from spleens of control mice demonstrated barely detectable levels for caspase 2, 3 and 8 mRNA, but caspase 1, 6, 7, 11 and 12 mRNA were not detected. The responses were essentially unchanged over the 42-day period. In contrast, transcripts for caspase 1, 2, 3, 6, 7, 8, 11 and 12 increased at 7 days PI and were even stronger at 14, 28 and 42 days PI in sPCV mice and at 42 days PI in mPCV mice. The enhanced mRNA expression was strongest for caspase 1, 2, 3 and 8 (Figure 3). Transcripts of caspase 14 were not upregulated.

### PCV2 increases mRNA of promoters and inhibitors of apoptosis in spleens of BALB/c mice

**Figure 4:**
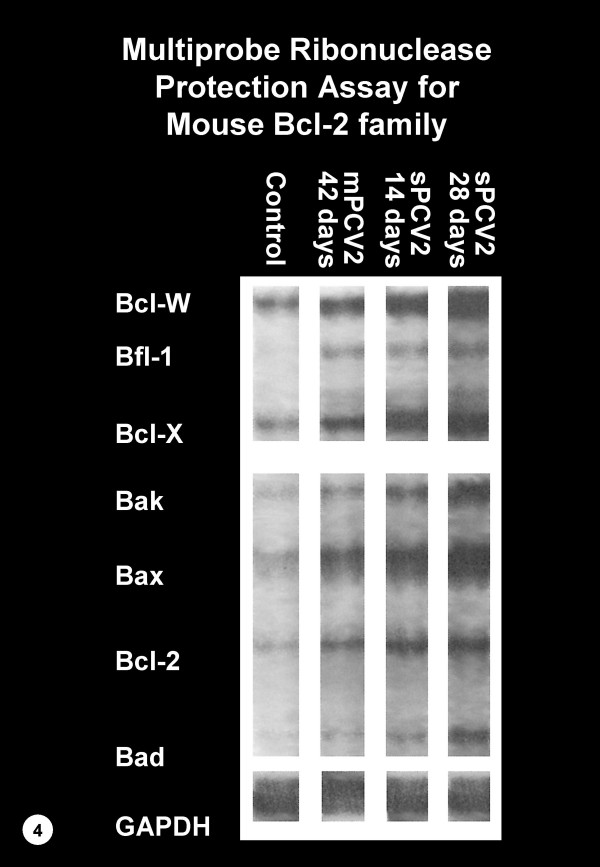
RNAse protection assay (RPA) for various bcl-2 homologue mRNAs from spleens of individual BALB/c mice. A representative RPA of a control mouse 28 days post sham inoculation is shown on the left. RPAs for representative BALB/c mice that were inoculated with a single (sPCV2) or multiple (mPCV2) doses of PCV2 and were euthanized at 14, 28 or 42 days PI, are shown on the right. Transcripts of apoptotic inhibitors bcl-2, bcl-w and bcl-X and apoptotic promoters bax, bak and bad are increased in sPCV and mPCV mice when compared to control mice. In addition, there was also upregulation of transcripts for bfl-1 in sPCV and mPCV mice. Quantitation of mRNA is identical for all samples.

Spleens of control mice had detectable levels of apoptosis inhibitors bcl-2, bcl-w and bcl-X mRNA and apoptosis promoters bax and bak mRNA. Bands for bcl-2 and bak were only faint. The responses were essentially unchanged over the 42-day period. In contrast, the transcripts of bcl-2, bcl-w, bcl-X, bax, bak and bad increased at 7 days PI and remained elevated at 14, 28 and 42 days PI in sPCV mice and at 42 days PI in mPCV mice. The expression of these transcripts was strongest in sPCV mice at 28 days PI. In addition, there was increased expression of bfl-1 mRNA in sPCV mice at 7, 14, 28 and 42 days PI and in mPCV mice at 42 days PI (Figure 4).

## Discussion

PCV2 caused apoptosis in spleens, lymph nodes and Peyer's patches of infected BALB/c mice, as confirmed by light and electron microscopic morphology [20] as well as by positive TUNEL assay and detection of activated caspase 3. Previously, PCV2 nucleic acid had been detected in the cytoplasm and rarely in the nucleus of putative histiocytes in follicular centers [20]. In serial sections of lymphoid tissue, putative histiocytes in follicular centers that were positive for activated caspase 3 were also positive for PCV2 nucleic acid. In contrast, no PCV2 nucleic acid was identified in apoptotic cells in the sinusoids of spleens from PCV2 infected mice.

Morphologic evidence of apoptosis was detected in PCV2 infected mice, but not in control mice that had been inoculated with tissue culture fluid. However, RPAs from spleens of control mice demonstrated barely detectable levels for caspase 3 mRNA. These data most likely represent some "basal" level of apoptosis involved in morphogenesis and homeostasis of lymphoid tissues that were not appreciated microscopically.

The mechanism of PCV2-induced apoptosis in mice is unknown. A possible mechanism could be the direct effect of a PCV2-encoded protein on virus infected and uninfected bystander cells. Viruses use various strategies to induce or inhibit apoptosis as a mechanism to enhance replication. Chicken anemia virus (ChAV), a member of the family of C*ircoviridae*, encodes a 14-kDa proline-rich protein referred to as apoptin that causes apoptosis in thymic lymphoblasts, intra- and extra-sinusoidal hemocytoblasts, and reticular cells. Expression of apoptin induces apoptosis in cultured transformed chicken mononuclear cells and in chicken thymocytes, both cells that are susceptible to infection with ChAV, but not in chicken embryo fibroblasts, which are not susceptible to infection with ChAV [34,35]. Apoptin also induces apoptosis in transformed and tumorigenic human cells in culture [34,35], but does not induce apoptosis in normal diploid human cells [9,50]. Apoptosis induced by apoptin is p53-independent, but requires activation of caspase 3 [7,8,37,51,52]. In normal cells, apoptin is localized mainly in the cytoplasm, whereas in transformed cells it is found in the nucleus [9]. A specific protein encoded by PCV2, similar to apoptin, could cause the detected apoptosis in lymphoid organs of PCV2 infected mice. So far, 11 potential open reading frames (ORF) have been identified in the genome of PCV2 [14] with six ORFs encoding putative proteins larger than 6 kDa. The function of the putative proteins encoded by the other ORFs 4 to 6 of PCV2 is unknown. Only ORF1 and ORF2 have been shown to encode the origin of replication and a replication-associated protein [26,27] and a structural protein [32], respectively. ORF2 of PCV2 shares a highly conserved basic N-terminal region that is similar to the major structural protein found in ChAV [30]. ORF3 has recently been characterized as a non-structural protein that is not essential for PCV2 replication in cultured PK15 cells and plays a major role in virus-induced apoptosis in cultured cells by activating initiator caspase-8 and effector caspase-3 pathways [24].

In an attempt to understand the mechanism of induction of apoptosis in the spleen of PCV2-infected BALB/c mice we investigated the regulation of mRNAs of various caspases and bcl-2 homologues using a multiprobe RNAse protection assay (RPA). Upregulation of caspase 1, 2, 3, 6, 7, 8, 11 and 12, but not 14 was detected. Among the 14 caspases that have been identified so far, caspases 3, 6 and 7 play a central role in driving the apoptotic effector pathway [23,43]. The expression of all three was increased in spleens of PCV2 infected mice. Caspases are produced as zymogens and have to be activated to initiate their function. In this study we confirmed activation of only caspase 3 in apoptotic macrophages using immunohistochemistry. Activated caspase 3 cleaves and inactivates the inhibitor for caspase-activated DNase (CAD), allowing CAD to enter the nucleus and degrade chromosomal DNA [12]. Activation of caspase 3 has been observed in various types of cells undergoing apoptosis induced by a variety of stimuli. In immune system-responsive cells, such as macrophages and lymphocytes, activation of caspase 3 has been shown to be required for apoptosis induced by Fas-FasL or TNF-α-TNFR interactions [43]. Because caspase 3 was the most significantly upregulated effector caspase and because it was detected in activated form in the cytoplasm of apoptotic macrophages, apoptosis in the spleens of PCV2 infected mice is likely mediated through the activation of caspase 3. Caspase 3 following initiation by caspase 8 has been shown to play a major role in the induction of PCV2-induced apoptosis in porcine cells [24]. In our studies transcripts for caspases 3 and 8 were both strongly elevated possibly suggesting a similar mechanism. Apoptosis in PCV2 infected porcine cells was not associated with initiator caspase 9. Unfortunately, caspase 9 was not included in the commercially available RPA, but should be tested in the future. The increased expression of the transcripts of apoptosis inhibitors bcl-2, bcl-w and bcl-X and apoptosis promoters' bax, bak and bad in the spleens of PCV2 infected mice, may indicate activation of an apoptotic pathway and attempts by the cells to prevent apoptosis. Based on samples of RNA extracted from whole tissue, it is impossible to conclude the specific pathway that led to apoptosis. In vitro experiments are required to further elucidate this mechanism.

In contrast to the other caspases, the primary functions of caspase 1 and 11 are generally believed to be proinflammatory [13,43]. Caspase 1 processes IFN-γ-inducing factor and regulates LPS-induced IFN-γ production. Activation of caspase 1, also referred to as IL-1β converting enzyme, in macrophages leads to cleavage of the precursor IL-1β into active IL-1 that may subsequently initiate an intense host inflammatory response. Therefore, it has been proposed that activation of caspase 1 converts a proapoptotic event into a proinflammatory one [53]. The strong upregulation of caspase 1 in this study correlated with upregulation of IL-1β mRNA in spleens of PCV2 infected mice (data not shown). We speculate that caspase 1 might play a role in inducing inflammation in lymphoid tissues of PCV2 infected mice.

Apoptosis has been confirmed in lymphoid tissues of PCV2 infected pigs [25,38]. However, it has been speculated, that lymphoid tissue depletion in PCV2-infected pigs is mainly related to decreased proliferative activity of lymphoid cells, and is caused by a long-standing absence of lymph node positive growth factors (mainly cytokines) produced by lymphocyte activation [41] rather than apoptosis [25]. In view of the knowledge on apoptosis in lymphoid tissues in pigs with PCV2 infection, the murine model presented here appears different from PMWS in pigs. PCV2 infected mice not only don't develop clinical signs and lesions typical of PMWS, but also exhibit an increase of apoptosis in lymphoid tissues, which does not seem to be the cause of lymphoid depletion in pigs.

## Methods

### Origin of tissues

Sixteen BALB/c mice (control mice) were sham inoculated and sixteen BALB/c mice were inoculated with a single (sPCV) dose of PCV2. In addition 4 BALB/c mice were inoculated with multiple (mPCV) doses of PCV2 6 times on days 0, 7, 14, 21, 28 and 35. Mice were inoculated intraperitoneally and intranasally with 0.2 ml of tissue culture fluid (control mice) or with 0.2 ml of PCV2 inoculum with a titer of 10^5 ^TCID_50_/1.0 ml (sPCV and mPCV mice). Four control and 4 sPCV mice were sacrificed 7, 14, 28 and 42 days post-inoculation (PI) and all 4 mPCV mice were sacrificed on day 42 PI. PCV2 infection was confirmed by PCR and in-situ hybridization and microscopic lesions as described [20]. Sections of tissue, including spleen, mediastinal and mesenteric lymph nodes, thymus and ileum were fixed in 10% buffered formalin for microscopic and immunohistochemical evaluation. Samples of spleen from each mouse were collected during necropsy and were frozen immediately in liquid nitrogen at -80°C for RNA extraction. This study met the standards of the Guide for the Care and Use of Laboratory Animals and the study protocol was approved by the Purdue Animal Care and Use Committee (PACUC #98–112).

### PCV2 detection

In-situ hybridization for demonstration of PCV2 nucleic acid was performed as previously described using a PCV2-specific oligoprobe [20]. Briefly, tissue sections were deparaffinized, digested with 0.25% pepsin and prehybridized. Hybridization was performed for 5 minutes at 105°C and 60 minutes at 37°C with a specific 3'-end digoxigenin labeled oligoprobe (5'-CCAACAAAATCTCTATACCC-3') at a concentration of 5 μl/1 ml using a commercial workstation (Fischer Scientific, Pittsburgh, PA). The detection system consisted of an anti-digoxigenin antibody (Boehringer Mannheim Biochemica, Indianapolis, IN) conjugated with alkaline phosphatase (dilution 1:500) and the substrates "NBT/X-Phos"(Nitro-blue tetrazolium/5-Bromo-4-chloro-3-indolylphosphate, Boehringer Mannheim Biochemica, Indianapolis, IN). Controls included lymphoid tissue from PCV2 infected pigs and sections of spleen and liver from PCV2 negative pigs and mice [19].

### Transmission electron microscopy

Samples of spleen from control and inoculated mice were fixed in 4% glutaraldehyde and postfixed by osmium tetroxide as previously described [20]. Tissues were embedded in epon and ultrathin sections were cut and stained with lead citrate and uranyl acetate. Sections were examined and photographed using a Joel JEM-1000CX transmission electron microscope.

### Cell death analysis

Following deparaffinization, selected sections of spleen, lymph nodes, thymus and ileum from control, sPCV and mPCV mice were incubated in target retrieval solution (Dako Corporation, Santa Barbara, CA) for 15 min at 95–99°C. Immunostaining was performed using the Dako autostainer (Dako Corporation, Santa Barbara, CA) by incubation with a polyclonal anti-mouse anti-activated caspase 3 antibody (R&D Systems, Minneapolis, MN) at a dilution of 1:100 at -4°C overnight, followed by a 1:500 dilution of a biotinylated goat anti-rabbit secondary antibody (Dako Corporation, Santa Barbara, CA). The antibody binding was localized using a peroxidase labeled streptavidin-biotin complex (Dako Corporation, Santa Barbara, CA) followed by diaminobenzidine as a chromogen substrate. After a final wash in automation buffer, the sections were counterstained with Lerner's hematoxylin. As negative controls, sections were incubated with isotype control antibodies.

For selected sections of spleen from control, sPCV and mPCV mice, DNA fragmentation was detected in situ by terminal deoxynucleotidyl transferase-mediated dUTP-DIG nick end labelening (TUNEL) using a commercial system (R&D Systems, 2000).

### RNAse Protection Assay (RPA)

Prior to analysis, total RNA was extracted from spleens of control, sPCV and mPCV mice that had been frozen at -80°C as described elsewhere [4]. Simultaneous detection and semiquantitation of bcl-2 homologues (mAPO-2) and caspase (mAPO-1) mRNAs were accomplished with the multiprobe RNase protection assay system from Pharmingen (San Diego, Calif). Briefly, a mixture of [^32^P]CTP-labelled antisense riboprobes was generated from bcl-2 homologues and caspase templates. These panels included templates for the murine housekeeping genes encoding L32 (a murine ribosomal protein) and glyceraldehyde-3-phosphate dehydrogenase (GAPDH, Young & Trowsdale, 1985), to ensure equal loading of total RNA onto the gels. A predetermined amount of total spleen RNA was hybridized overnight at 56°C with 300 pg of the ^32^P-antisense riboprobe mixture. After hybridization, the samples were digested with 2,500U of T_1 _nuclease (Gibco-BRL, Gaithersburg, Md.). Nuclease-protected RNA fragments were purified by ethanol precipitation. After purification, the samples were resolved on a 4.5% polyacrylamide sequencing gel. Protected bands were observed after exposure of the gels to Fuji X-ray film (Fisher, Itasca, Ill.). The specific bcl-2 homologues and caspase bands were identified on the basis of their individual migration patterns in comparison with the undigested probes. The intensities of the bands from the mRNA of spleens of sPCV and mPCV mice were compared to those of uninfected control spleens.

RPAs of each panel of bcl-2 homologues and caspase mRNAs were performed at least 3 times with similar results for samples of spleen from control, sPCV and mPCV mice.

## Authors' contributions

MK designed and coordinated the study, carried out TEM and PCV detection and drafted the manuscript, GWS participated in the study design, coordination and interpretation, SKM participated in study design and interpretation, AN carried out the RPAs, EJG carried out the cell death analysis, HH participated in study design and interpretation. All authors read and approved the final manuscript
